# Cellular immunity reflects the persistent symptoms among COVID-19 recovered patients in Japan

**DOI:** 10.1038/s41598-023-35505-w

**Published:** 2023-07-08

**Authors:** Yoshiharu Miyata, Kohjin Suzuki, Tatsuya Nagano, Keiji Iida, Takehiro Hasegawa, Hitoshi Uga, Hiroshi Matsuoka

**Affiliations:** 1 Division of Bioresource Research and Development, Department of Social/Community Medicine and Health Science, Kobe University Graduate School of Medicine, 1-5-1 Minatojimanakamachi, Chuo-ku, Kobe, 650-0047 Japan; 2 Division of Respiratory Medicine, Department of Internal Medicine, Kobe University Graduate School of Medicine, 7-5-1 Kusunoki-cho, Chuo-ku, Kobe, 650-0017 Japan; 3 Division of Diabetes and Endocrinology, Hyogo Prefectural Kakogawa Medical Center, 203, Kanno, Kanno-cho, Kakogawa, Hyogo 675-8555 Japan; 4 System Technologies Laboratory, Sysmex Corporation, 4-4-4 Takatsukadai, Nishi-ku, Kobe, 651-2271 Japan; 5 Central Research Laboratories, Sysmex Corporation, 4-4-4 Takatsukadai, Nishi-ku, Kobe, 651-2271 Japan; 6 Research and Development Division, Sysmex R&D Centre Europe GmbH, Falkenried 88, 20251 Hamburg, Germany

**Keywords:** Infectious diseases, Infectious diseases, Lymphocytes

## Abstract

Coronavirus disease (COVID-19) often causes persistent symptoms long after infection, referred to as “long COVID” or post-acute COVID-19 syndrome (PACS). This phenomenon has been studied primarily concerning B-cell immunity, while the involvement of T-cell immunity is still unclear. This retrospective study aimed to examine the relationship among the number of symptoms, cytokine levels, and the Enzyme-linked immunosorbent spot (ELISPOT) assay data in patients with COVID-19. To examine inflammatory conditions, plasma interleukin (IL)-6, IL-10, IL-18, chemokine ligand 9 (CXCL9), chemokine ligand 3 (CCL3), and vascular endothelial growth factor (VEGF) levels were analyzed using plasma obtained from COVID-19 recovery patients and healthy controls (HC). These levels were significantly higher in the COVID-19 group than those in the HC group. ELISPOT assays were performed to investigate the correlation between COVID-19 persistent symptoms and T-cell immunity. Cluster analysis of ELISPOT categorized COVID-19 recovery patients in the ELISPOT-high and -low groups, based on the values of S1, S2, and N. The number of persistent symptoms was significantly higher in the ELISPOT-low group than those in the ELISPOT-high group. Thus, T cell immunity is critical for the rapid elimination of COVID-19 persistent symptoms, and its measurement immediately after COVID-19 recovery might predict long-term COVID-19 or PACS.

## Introduction

Severe acute respiratory syndrome coronavirus 2 (SARS-CoV-2) has caused a pandemic as a new type of coronavirus disease (COVID-19). Despite the resolution of symptoms and infectivity in COVID-19, various symptoms persist from the acute phase or new or reemerging, and persistent symptoms are reported during the disease^1–3^, regardless of the severity at diagnosis. The persistence of symptoms, including fatigue, dyspnea, cough, dysgeusia, or alopecia over several months is termed as “long COVID” and has led to the proposed diagnosis of post-acute COVID-19 syndrome (PACS).

The immune system of some patients infected with SARS-CoV-2 are unable to clear the virus for long periods, and it may persist in certain organs or tissue reservoirs after acute infection, affecting immunity and causing chronic symptoms^4^. The relationship between T-cell immunity and COVID-19 has also received particular attention, and a correlation between the duration of symptoms and antigen-specific T-cell responses to SARS-CoV-2 spike protein have been reported^5^. Thus, a T-cell-based assay to examine the lifespan of the immune response after SARS-CoV-2 infection or vaccination may be an important COVID-19 indicator^6^.

Enzyme-linked immunosorbent spot (ELISPOT) assay can detect and quantify antigen-specific cytokine-producing cells in peripheral blood by measuring interferon (IFN)-γ released from antigen-specific T cells stimulated with pathogen-specific peptides using enzyme-linked immunosorbent assay^7^. The ELISPOT assay for SARS-CoV-2 has a potential clinical value similar to the QuantiFERON-TB Gold test for TB diagnosis, although its clinical significance has not yet been established^8^.

This study aimed to examine the ELISPOT reactivity to Japanese patients who have recovered from COVID-19, as well as to examine the relationship among the number of symptoms, cytokine levels, and the ELISPOT assay data in patients with COVID-19. To achieve this, we performed an ELISPOT assay using peripheral blood mononuclear cells (PBMC) obtained from patients recovering from COVID-19 and found that the ELISPOT assay count was low in patients with high symptom counts. Furthermore, cytokine and chemokine assays using plasma revealed that interleukin (IL) –10, chemokine (C–X–C motif) ligand 9 (CXCL9), chemokine (C–C motif) ligand 3 (CCL3), and vascular endothelial growth factor (VEGF) levels remained elevated.

## Results

### There was no significant relation between COVID-19 severity and persistent symptoms

**Figure 1:**
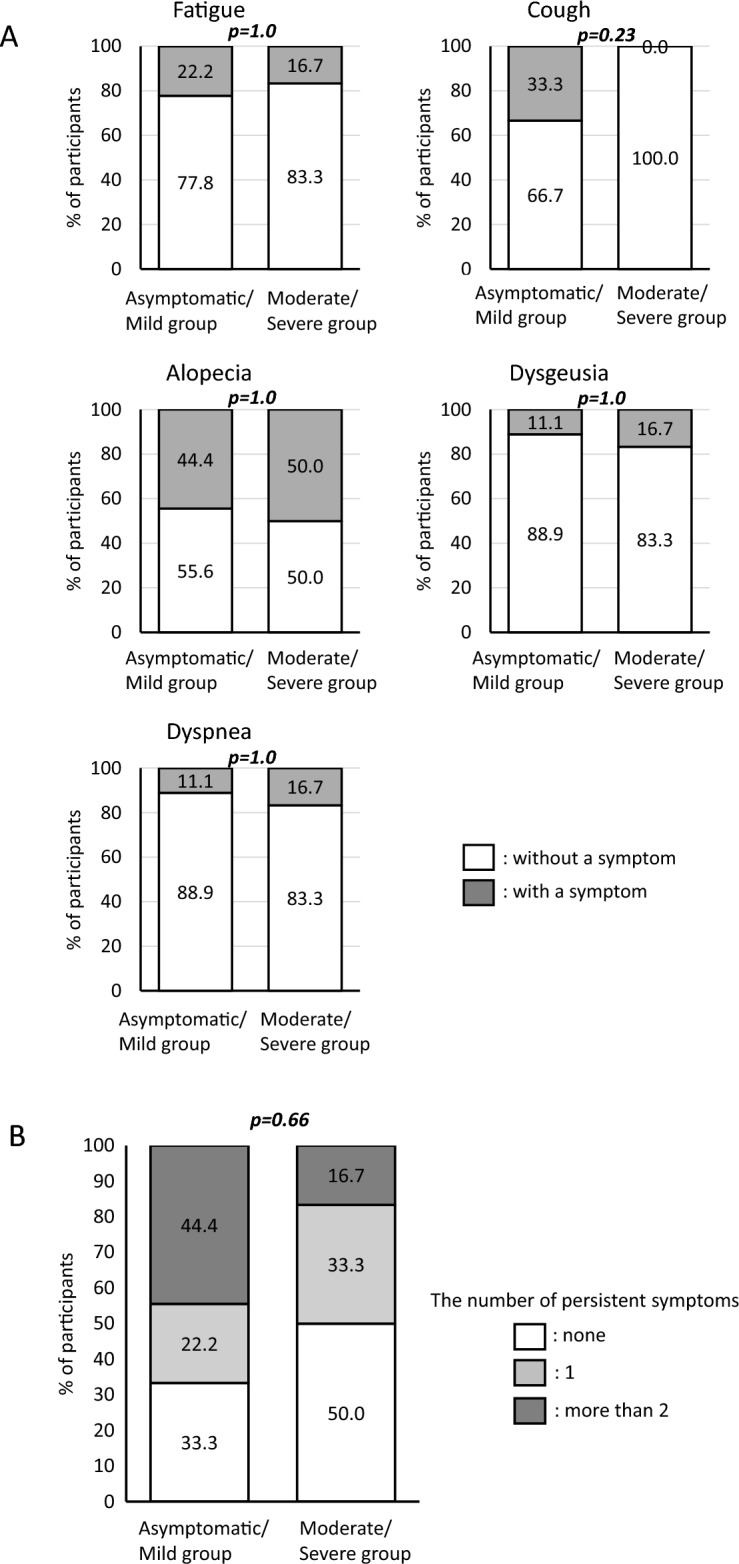
(**A**) Persistent symptoms of participants recovered from COVID-19. The percentages of participants who had prolonged symptoms of fatigue, cough, alopecia, dysgeusia, and dyspnea were shown. Each persistent symptoms were compared between Mild; asymptomatic or mild patients at diagnosis (N = 9), and Severe; moderate to severe patients at diagnosis (n = 6). The white bar shows the percentages of the patients without persistent symptoms, and the gray bar shows the percentages of the patients who had persistent symptoms. P-values were tested by fisher’s exact test. (**B**) The number of persistent symptoms were compared between two groups. The white, gray, and dark gray bars represents the number of persistent symptoms of none, one, and more than two, respectively. P-values were tested by fisher’s exact test.

Fifteen COVID-19 recovery patients and ten healthy controls participated in this study. To consider the factors associated with persistent COVID-19 symptoms, we first investigated whether COVID-19 severity affected persistent symptoms. Table 1 shows the Common Terminology Criteria for Adverse Events (CTCAE) scores of the five representative symptoms (fatigue, cough, alopecia, dysgeusia, and dyspnea) that persisted for at least 1 month after recovery in each patient. The patients reported no symptoms other than those listed above. We compared the five persistent symptoms with COVID-19 severity. Statistical analysis did not reveal significant differences in the frequency of fatigue, alopecia, dysgeusia, or dyspnea among the asymptomatic, mild, and moderate/severe groups (Fig. 1A,  P = 1.0 for all, respectively). All patients with persistent cough were in the asymptomatic/mild group, although the difference was not statistically significant (P = 0.23). We also examined the number of persistent symptoms between the asymptomatic/mild, and moderate/severe groups, and found no significant differences between two groups (Fig. 1B, P = 0.66). No significant difference in symptom duration was found between two groups (Supplementary Fig. 1, P = 1.0). These results suggest that COVID-19 severity does not directly affect persistent COVID-19 symptoms.

**Table 1:** Persistent symptoms of participants recovered from COVID-19.

		CTCAE 5 grade, (persistent period, month)				
Participant ID	Severity at diagnosis	Fatigue	Cough	Alopecia	Dysgeusia	Dyspnea
01	Asymptomatic	1 (12 M)	0	1 (6 M)	0	0
02	Asymptomatic	0	2 (3 M)	1 (3 M)	0	0
03	Mild	1 (9 M)	0	0	0	0
04	Mild	0	1 (1 M)	1 (3 M)	0	1 (1 M)
05	Mild	0	0	1 (3 M)	0	0
06	Mild	0	1 (1 M)	0	1 (1 M)	0
07	Mild	0	0	0	0	0
08	Mild	0	0	0	0	0
09	Mild	0	0	0	0	0
10	Moderate	0	0	1 (6 M)	0	0
11	Moderate	0	0	0	0	0
12	Moderate	1 (3 M)	0	1 (3 M)	1 (12 M)	2 (6 M)
13	Moderate	0	0	0	0	0
14	Moderate	0	0	0	0	0
15	Severe	0	0	1 (3 M)	0	0

Table 2 shows the demographic and clinical characteristics of the COVID-19 recovered patients analyzed in this study. Clinical records showed that D-dimer levels were higher in the moderate/severe group than those in the asymptomatic/mild group (P = 0.0021 by Mann–Whitney *U* test). No significant differences were found in the other clinical parameters. Although reduction in antibody titers and ELISPOT reactivity over time after infection have been reported, the median time from diagnosis to blood collection was 39 days in the asymptomatic and mild groups, and 46 days in the moderate/severe group, with no statistically significant differences (P = 0.32). Previous studies reported activation of both innate and adaptive immune system lead to the elimination of infected cells, and elevation of proinflammatory cytokines was observed in COVID-19 patients^9^. We thought SARS-CoV-2 specific antibodies, SARS-CoV-2 specific T cells, and inflammatory cytokines, are important factors involved in the pathophysiology of COVID-19. Therefore, we aimed to identify which of these three factors was most closely associated with persistent COVID-19 symptoms.

**Table 2:** Participants’ demographics at diagnosis of COVID-19.

	Asymptomatic/mild (n = 9)	Moderate/severe (n = 6)	*p* value
	Median (Q1–Q3)		
Age (yrs)	47 (45–59)	66.5 (58.8–73.5)	*p* = 0.16
Male/female	4/5	3/3	
WBC (/uL)	4610 (4380–5657.5)	4645 (4195–5095)	*p* = 0.37
Ly (%)	24 (22.6–27.8)	16.6 (15–21.4)	*p* = 0.26
Ly (/uL)	1242.2 (1144.8–1336.8)	762.75 (661.5–918.9)	*p* = 0.20
LDH (U/L)	197 (167.8–208)	357.5 (302.5–436.5)	*p* < 0.05
CRP (mg/L)	0.22 (0.19–0.75)	3.65 (2.11–7.75)	*p* = 0.12
D-Dimer (ug/mL)	0.5 (0.45–0.6)	1.3 (1.1–2.2)	*p* < 0.05
Medication			
No treatment	9/9	0/6	
Favipiravir	0/9	2/6	
Ritonavir	0/9	1/6	
Ciclesonide	0/9	3/6	
Antibiotics	0/9	1/6	
Sampling (days after diagnosis)	39 (37.5–42.3)	46 (41.5–99.3)	*p* = 0.32

Plasma SARS-CoV-2-specific antibody levels, SARS-CoV-2-specific effector/memory T-cell responses, and some plasma inflammatory cytokines were significantly higher in Japanese COVID-19 recovered patients than those in healthy participants.

**Figure 2:**
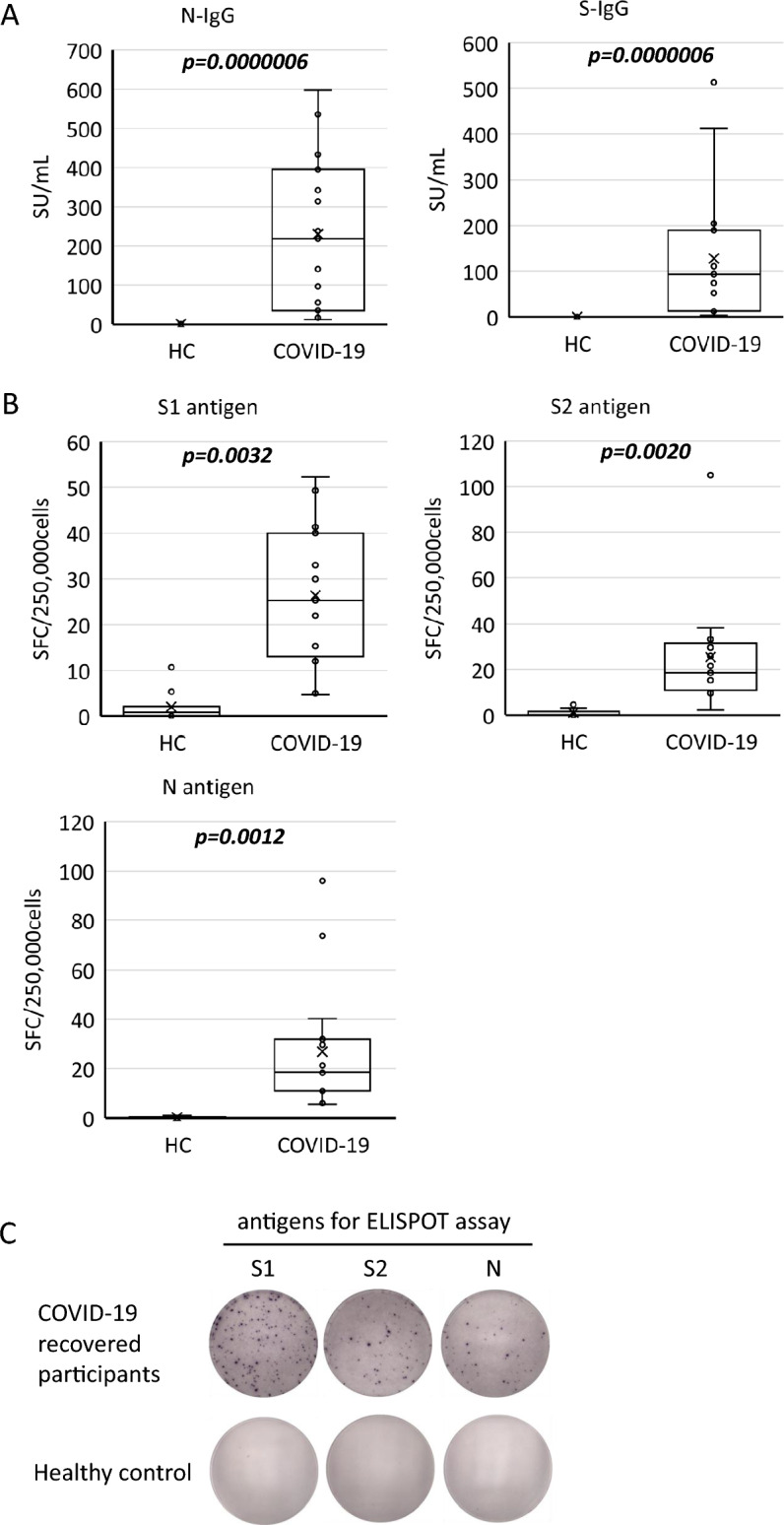
Plasma IgG levels and the number of SARS-CoV-2 specific effector/memory T-cells of the participants at recovery period of COVID-19. (**A**) Plasma N-IgG and S-IgG level, (**B**) the number of SARS-CoV-2 specific effector/memory T-cells measured by ELISPOT assay, and (**C**) the representative spot images of ELISPOT assay. Each parameters were compared between HC; healthy control (n = 10) and COVID-19; COVID-19 recovered participants (n = 15). P-values were tested by the Mann–Whitney *U* test. *SU* sysmex unit, *SFC* spot forming cells.

Further, we examined the plasma SARS-CoV-2 S-immunoglobulin G (IgG) and N-IgG levels to confirm no history of infection in the healthy volunteers and that all the COVID-19 recovered patients had acquired immunity to SARS-CoV-2. As a result, both SARS-CoV-2 S-IgG and N-IgG levels were significantly higher in the COVID-19 recovered patients’ group than those in the healthy control (HC) group (Fig. 2A,  P = 0.0000006, 0.0000006, respectively).

We then examined SARS-CoV-2 specific effector/memory T-cell responses using the ELISPOT assay kit provided by Oxford Immunotec. IFN-γ spots produced by SARS-CoV-2 antigen-specific T-cells were significantly increased by the stimulations of SARS-CoV-2 specific S1, S2, and N antigen peptides in the COVID-19 group compared to those in the HC group (Fig. 2B, P= 0.0032, 0.0020, 0.0012, respectively). Typical images of the ELISPOT assay from COVID-19 recovered patients in Japan are shown in Fig. 2C.

**Figure 3:**
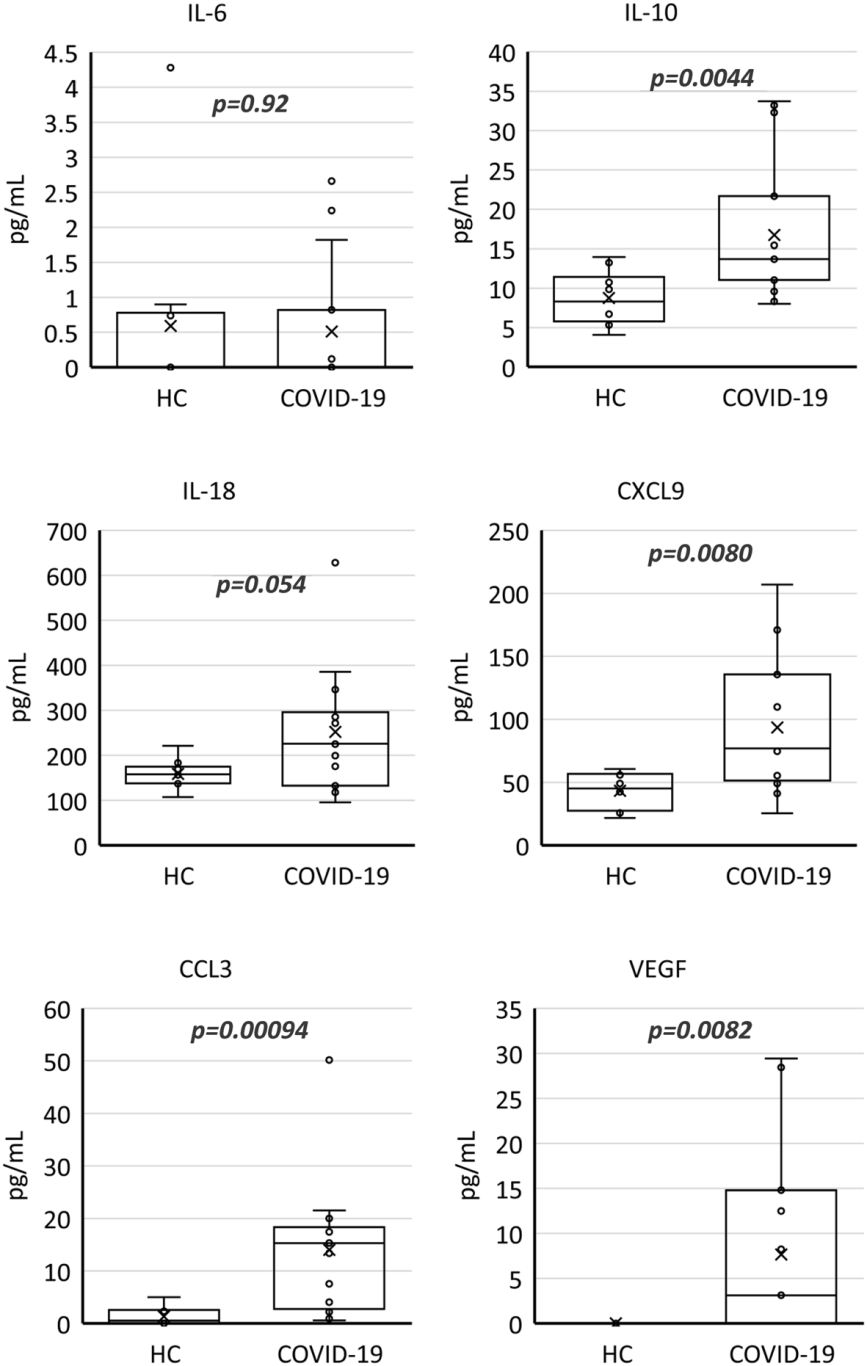
Plasma IL-6, IL-10, IL-18, CXCL9(MIG), CCL3(MIP-1a) and VEGF levels of the participants at recovery period of COVID-19. Each cytokines were compared between HC; healthy control (n = 10) and COVID-19; COVID-19 recovered participants (n = 15). P-values were tested by the Mann–Whitney *U* test.

To examine the inflammatory conditions of COVID-19 recovered patients, plasma IL-6, IL-10, IL-18, CXCL9, CCL3, and VEGF levels were measured, and these parameters were compared between the COVID-19 recovered and healthy control groups. Interestingly, IL-10, CXCL9, CCL3, and VEGF levels were significantly higher in the COVID-19 group than those in the HC group. (Fig. 3, P = 0.0044, 0.0080, 0.00094, 0.0082, respectively). There were no significant differences in IL-6 and IL-18 levels between the two groups (P = 0.92, 0.054, respectively).

These results confirm that high levels of SARS-CoV-2 specific antibodies and SARS-CoV-2 specific T cells were maintained even after COVID-19 recovery. Interestingly, it was also found that some plasma inflammatory cytokine levels were still higher in the patient group than those in healthy volunteers, suggesting differences in physiological conditions between healthy and COVID-19 recovered patients.

### The combined counts of the S1, S2, and N antigens in the ELISPOT assay were associated with the number of symptoms

**Figure 4:**
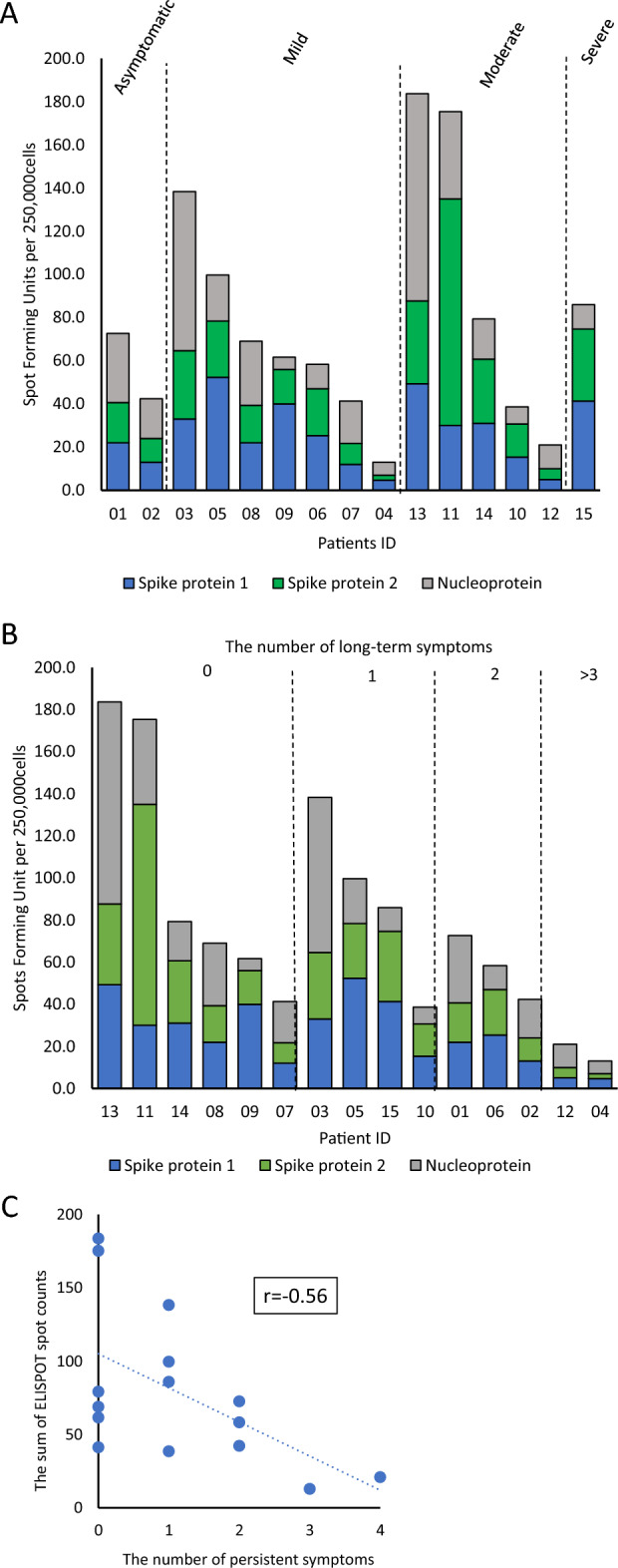
Magnitude of IFN-γ T cell responses measured by ELISPOT assay for each individual. The vertical axis shows the sum of ELISPOT counts for S1, S2 and N antigens. Hortizontal axis shows individual cases in order of severity of COVID-19 (**A**). Horizontal axis shows cases sorted by number of COVID-19 symptoms (**B**). Correlation between the sum of ELISPOT spots in panel 1, 2 and 3 and the number of persistent symptoms. Correlation efficiency was calculated by Spearman’s rank correlation coefficient (**C**).

First, we examined the combined spot counts of the S1, S2, and N antigens and the severity of COVID-19 in individual cases; however, a clear trend could not be established (Fig. 4A). Next, we examined the combined counts of the S1, S2, and N antigens in the ELISPOT assay and the number of persistent symptoms. It was found that the lower the ELISPOT count, the higher were the number of symptoms, with r = − 0.56 by Spearman’s rank correlation coefficient (Fig. 4B,C). Since the ELISPOT assay reflects memory T-cell response^10^, our results suggest that patients with COVID-19 showing more persistent symptoms may have a poor memory T-cell response in subsequent months.

### SARS-CoV-2 specific T-cell responses were related to long-term COVID-19 symptoms

To further investigate whether the persistent symptoms of COVID-19 are associated with our findings of antibody titers, cytokine measurements, and ELISPOT levels, we performed a hierarchical cluster analysis.

**Figure 5:**
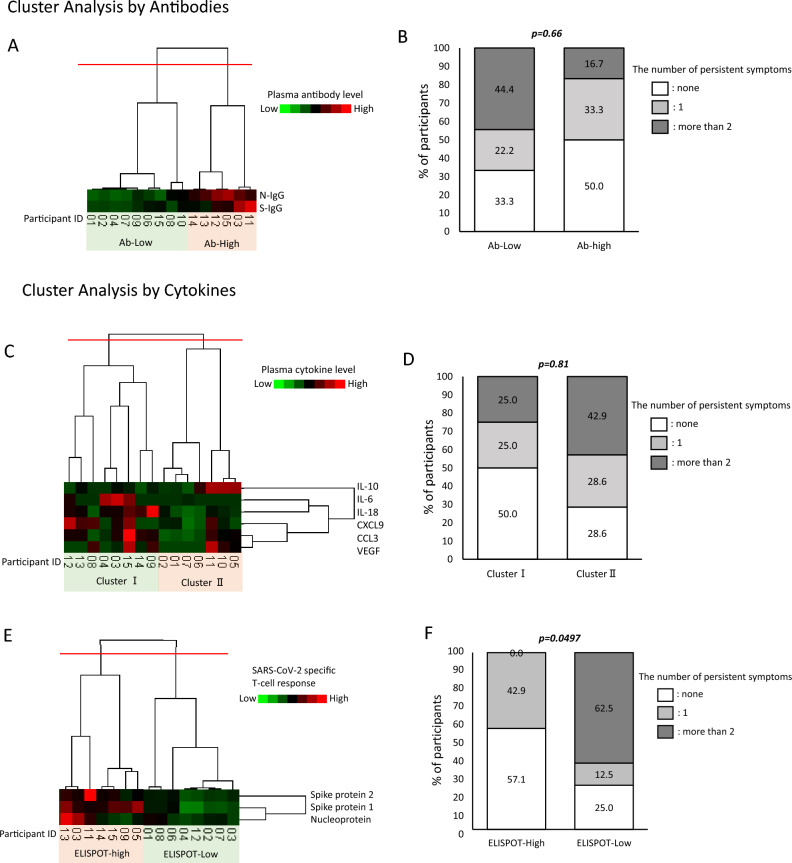
Cluster analysis of the participants by (**A**) plasma S-IgG and N-IgG levels, (**C**) plasma cytoline levels, and (**E**) SARS = CoV-2 specific T-cell responses. The participants were classified into two groups, (**A**) Ab-low group (n = 9) and Ab-high group (n = 6). (**B**) Cluster I (n = 8) and cluster II (n = 7). (**C**) ELISPOT-High group (n = 7) and ELISPOT-Low group (n = 8). (**B**,**D**,**F**) The frequency of persistent symptoms between these two groups were compared. The white bar shows the percentages of the patients without persistent symptoms the light gray bar shows the percentages of the patients who has one persistent symptoms, and the dark gray bar shows the percentages of the patients who has more than two persistent symptoms. P-values were tested by Fisher’s exact test.

We first stratified the COVID-19 recovered patient group into the following two groups based on cluster analysis of antibody levels: Ab-high and Ab-low group (Fig. 5A). No significant association was found between the number of persistent symptoms in these groups (Fig. 5B, P = 0.66). Further, based on the six plasma cytokine levels measured in this study, the COVID-19 group was classified into two groups, Clusters I and II (Fig. 5C); the number of persistent symptoms did not differ significantly between the two groups (Fig. 5D, P = 0.81).

Finally, we classified the patients into two groups, ELISPOT high and low, based on ELISPOT S1, S2, and N values (Fig. 5E), and found that the number of persistent symptoms was significantly higher in the ELISPOT-**l**ow group than those in the ELISPOT-high group (Fig. 5F, P = 0.0497). These results suggest that patients with COVID-19 having insufficient T-cell immunity to SARS-CoV-2 after recovery are more likely to have persistent COVID-19 symptoms. In addition, although no statistically significant differences were found in the duration of symptoms between two groups, the symptoms were relatively more persistent in the ELISPOT-low group than those in the ELISPOT-high group (Supplementary Fig. 2, P = 0.25). Moreover, there were no significant differences of days from diagnosis to the blood sampling between ELISPOT-high and -low group (Supplementary Fig. 3, P = 0.61).

## Discussion

The mechanisms of long-COVID and PACS have been studied primarily concerning B-cell immunity, while the involvement of T-cell immunity still needs further investigation^8^. In this study, we used the “T-Spot Discovery kit” to analyze antigen responses (S1, S2, and N) and a fully automatic immune analyzer to analyze the corresponding antibody titers (anti-S and anti-N). In a comparison of patients treated for SARS-CoV-2 infection and healthy controls, plasma N-IgG, S-IgG titers, and ELISPOT reactivity against S1, S2, and N antigens were significantly elevated in the patient group.

In a survey of patient groups, no significant differences were found in the number of symptoms among the symptom groups (asymptomatic, mild, and moderate/severe group). Cluster analysis showed no correlation between the number of symptoms and antibody or cytokine levels; however, ELISPOT analysis of the high and low groups (based on S1, S2, and N values) revealed a trend towards multiple symptoms in the low group. Since the ELISPOT test evaluates the activation potential of INF-γ-producing memory T cells, our results suggest that SARS-CoV-2 antigen-specific memory T cells are more likely to be activated in patients with fewer symptoms compared to in those with more symptoms.

Several previous studies have reported the use of ELISPOT in evaluating patients with COVID-19 compared ELISPOT-positive patients with positive serological tests in immunized (n = 15) and non-immunized (ELISPOT-negative, n = 15) groups and found marginal differences in the characteristics or clinical features at diagnosis. Clinical characteristics of patients with persistent symptoms at a median of 174 days after symptom onset were also similar to age-matched COVID-19 convalescent patients with no persistent symptoms^11^. Most other ELISPOT studies have been performed by collecting PBMCs from recovered patients. Peng et al. performed ELISPOT using PBMC from patients recovered from COVID-19 using a panel of 423 overlapping peptides and reported that the T-cell response was stronger in more severely ill patients^12^. Another report of ELISPOT for spike protein, nucleoprotein, membrane protein, and E/ORF found no clear correlation between disease severity and total spot count^13^. However, this report did not examine correlations based on the number of symptoms of patients, as performed in our study.

Here, we found no clear relationship between disease severity and the combined ELISPOT counts of S1, S2, and N. In contrast, our results suggest that more symptomatic patients tend to have lower combined ELISPOT counts of S1, S2, and N. This indicates that post-infection patients with multiple symptoms have poor memory T cell activation. SARS-CoV-2 can infect a wide range of cell types in various human organs^14^. Thus, patients with less reactive T cell lymphocytes may present more diverse symptoms. Therefore, we can hypothesize that patients with poor T-cell activation will have more symptoms early in disease onset.

The present study also analyzed plasma cytokines and chemokines to assess the state of immune balance after recovery, focusing on IL-18, CXCL9, and CCL3 as Th1 cytokines, IL-6 and IL-10 as Th2 cytokines, and VEGF as a fibrosis marker; significant results have been observed earlier in the analyses of plasma from patients with COVID-19 in the acute phase of infection^15^.

The levels of IL-10, CXCL9, CCL3, and VEGF were significantly elevated in the patient group compared to those in the healthy group, while IL-6 and IL-18 levels showed no significant differences. The results are notable, since, to date, although there have been many reports of cytokines associated with acute critical illness, few reports have evaluated cytokines after recovery. The pro-inflammatory cytokines IL-6 and IL-18 were not significantly different in the disease group compared to those in the healthy group, suggesting that acute inflammation subsided while COVID-19 was cured.

IL-10 is an immunomodulatory cytokine which downregulates the activated immune responses. It is secreted by various cells such as Th2 and Treg cells. Generally, IL-10 production is inhibited in Th1 cells; however, studies in mice have shown that Th1 cells acquire plasticity and start secreting IL-10 under chronic antigen stimulation^16^. CXCL9 acts via the agonistic receptors CXCR3 and CCL3 via CCR5^17^, inducing memory T cells in the lymph nodes to migrate to inflamed tissues by antigen presentation from dendritic cells, leading to differentiation and activation of Th1-type T lymphocytes^18^. Therefore, our cytokine assay results suggest that chronic inflammation of the Th1 system due to antigen stimulation may persist in the patient’s body after COVID-19 recovery. VEGF is abundant in the lungs and originates from the alveolar epithelium and macrophages; however, its function is unknown. VEGF may be a protective factor in acute respiratory distress syndrome and tends to increase with the severity of idiopathic pulmonary fibrosis^19^. Therefore, high VEGF levels may reflect persistent chronic inflammation in the lungs.

Recently, mathematical models indicate that small amounts of the virus can persist in the body, which may result in prolonged COVID and PACS, and SARS-CoV-2 infection decreases dendritic cell counts regardless of severity^14^. This report also seems highly consistent with the results of the present study. Thus, patients with more diverse symptoms have fewer dendritic cells, lower T-cell reactivity, and lower ELISOT. This may also explain the high cytokine levels that reflect the chronic inflammation caused by prolonged and persistent SARS-CoV-2 infection.

This study has several limitations. First, we evaluated a small number of COVID-19 recovery patients. In addition, data on the number and duration of COVID-19 symptoms were collected retrospectively through interviews and were based on patient memory. We selected persistent symptoms for analysis, focusing on those that were reported by at least one patient. While no patients reported persistent symptoms except for the five chosen ones in this analysis, numerous other symptoms have been reported elsewhere and thus further investigation is required.

All patients and healthy controls in the present study were unvaccinated, and it can be assumed that results may differ under the current situation where majority of the population is vaccinated. Kato et al. reported that the ELISPOT assay is useful for measuring vaccine-induced cellular immunity against SARS-CoV-2^20^; thus, the ELISPOT assay was expected to be useful for confirming vaccine efficacy. Similarly, cytokines may be useful biomarkers of chronic inflammation resulting from chronic SARS-CoV-2 infection. These measurement assays should be validated in larger prospective studies.

In conclusion, our results suggest that T-cell immunity plays an important role in the prompt elimination of COVID-19 persistent symptoms. In addition, from a clinical point of view, the measurement of T cell immunity after COVID-19 recovery might be useful for the assessment of long periods of COVID-19 or PASC, which will allow prescribing further appropriate treatment as needed.

## Materials & methods

### Clinical data & sample collection

All patients in the study were adults (aged 20 years or older), and written informed consent was obtained from each individual. Patients who participated in the study were diagnosed with COVID-19 using a SARS-CoV-2 specific polymerase chain reaction test^21^ or antigen testing and recovered from COVID-19 after treatment. Healthy participants without COVID-19 pseudo-symptoms (fever, respiratory distress, upper respiratory tract symptoms, or abnormal chest shadows) were recruited as the control group. The exclusion criteria for both the groups were as follows: diagnosis with an autoimmune disease; receiving treatment with antibody drugs, immune checkpoint inhibitors, or immunosuppressive drugs (e.g. methotrexate or steroids); and judged as ineligible for unforeseen reasons at the time of study designing, such as a patient who was difficult to understand this study because of severe dementia. We classified the severity of COVID-19 into four categories (asymptomatic, mild, moderate, and severe) according to the previous report^22^, and evaluated the patients in two groups: asymptomatic/mild and moderate/severe.

All patients were unvaccinated at the time of blood collection. Whole blood samples from ten healthy volunteers were obtained from Kobe University Hospital as the control group.

Whole blood samples from one COVID-19 recovered patient were obtained from Kobe University Hospital and 14 patients from Hyogo Prefectural Kakogawa Medical Center. Two of these patients were asymptomatic, seven had mild symptoms, five had moderate symptoms, and one had severe symptoms (Table 1). These patients showed no clinical symptoms of COVID-19 2 weeks prior to blood sampling. Clinical information at the time of diagnosis was obtained from medical records. All the samples were collected in heparin-blood collection tubes.

This study complied with the requirements of the Declaration of Helsinki and was approved by the Medical Research Ethics Committee of Kobe University Hospital (B2056705) and Sysmex Corporation (2020-206). Informed consent was obtained from all participants.

### Telephone survey on persistent COVID-19 persistent symptoms

Approximately a year after blood sampling, a telephone survey was conducted to investigate the persistent symptoms of patients who recovered from COVID-19. The presence or absence and duration of five persistent symptoms including fatigue, cough, alopecia, dysgeusia, and dyspnea were surveyed and evaluated using the CTCAE version 5.0. These symptoms were determined by the attending physician, based on complaints from patients when he was engaged in the outpatient treatment of the subject patients. In addition, we also checked whether there were any subjective symptoms other than these five symptoms when the telephone interview was conducted.

### ELISPOT assay

Whole blood samples were diluted using Roswell Park Memorial Institute Medium 1640 (Thermo Fisher Scientific, Waltham, MA, USA), and PBMCs were isolated using Ficoll Paque Plus (GE Healthcare Bioscience, Piscataway, NJ, USA) and Leucosep Tube (Greiner Bio-one, Frickenhausen, Germany). PBMC isolation was performed within 32 h of blood sampling, and T-cell Xtend (Oxford Immunotec, Oxford, UK) was added before isolation.

The ELISPOT assay was performed using T-SPOT Discovery (Oxford Immunotec). Cells (2.5 × 10^5^) were diluted using AIM-V medium (Thermo Fisher Scientific) and stimulated by three kinds of SARS-CoV-2 specific antigen peptide pools (spike glycoprotein S1 subunit; S1, spike glycoprotein S2 subunit; S2, and nucleocapsid protein; N antigen) for 20 h. The amino acid sequences of the S1, S2, and N antigens have not been previously published. After the assay, IFN-γ-producing cells were counted as spots under a ImmunoSpot Analyzer (Cellular Technology Limited, Cleaveland, OH, USA).

### Serology testing

Diluted plasma samples obtained when isolating PBMCs for the ELISPOT assay were used for serological testing. Plasma levels of SARS-CoV-2-specific S-IgG, N-IgG, cytokines, and chemokines, including IL-6, IL-10, IL-18, CXCL9, CCL3, and VEGF, were measured using a fully automatic immune analyzer (HISCL-5000; Sysmex Corp., Hyogo, Japan), according to previous reports^15,23^.

### Statistical analyses

In boxplots, values were expressed with the 10th, 25th, 50th, 75th, and 90th percentiles, mean values were expressed with x, and single values were superimposed on the boxplots. For statistical analyses, the Mann–Whitney *U* test was used to compare the differences between the two experimental groups. Fisher’s exact test was used to examine the significance of the association between the classifications. All statistical tests were performed nonparametrically due to the small sample size. All statistical tests were two-tailed, and statistical significance was set at P < 0.05. Statistical analyses were performed using EZR based on R and R commanders^24^. Unsupervised hierarchical cluster analysis was performed using Cluster 3.0 (University of Tokyo Human Genome Center). Cluster analysis was performed using complete linkage based on Euclidean distance.

## Supplementary Information

Supplementary Figures.

## Data Availability

All data associated with the experiments is available in the supporting information.
