# Supplementary material for: Cellular immunity reflects the persistent symptoms among COVID-19 recovered patients in Japan
Source: Sci Rep. 2023 Jul 8;13:11071. doi: 10.1038/s41598-023-35505-w (PMC10329673; doi:10.1038/s41598-023-35505-w)
Supplement: Supplementary file 1 — Supplementary Figures. [file 41598_2023_35505_MOESM1_ESM.pdf]

Supplemental figure 1

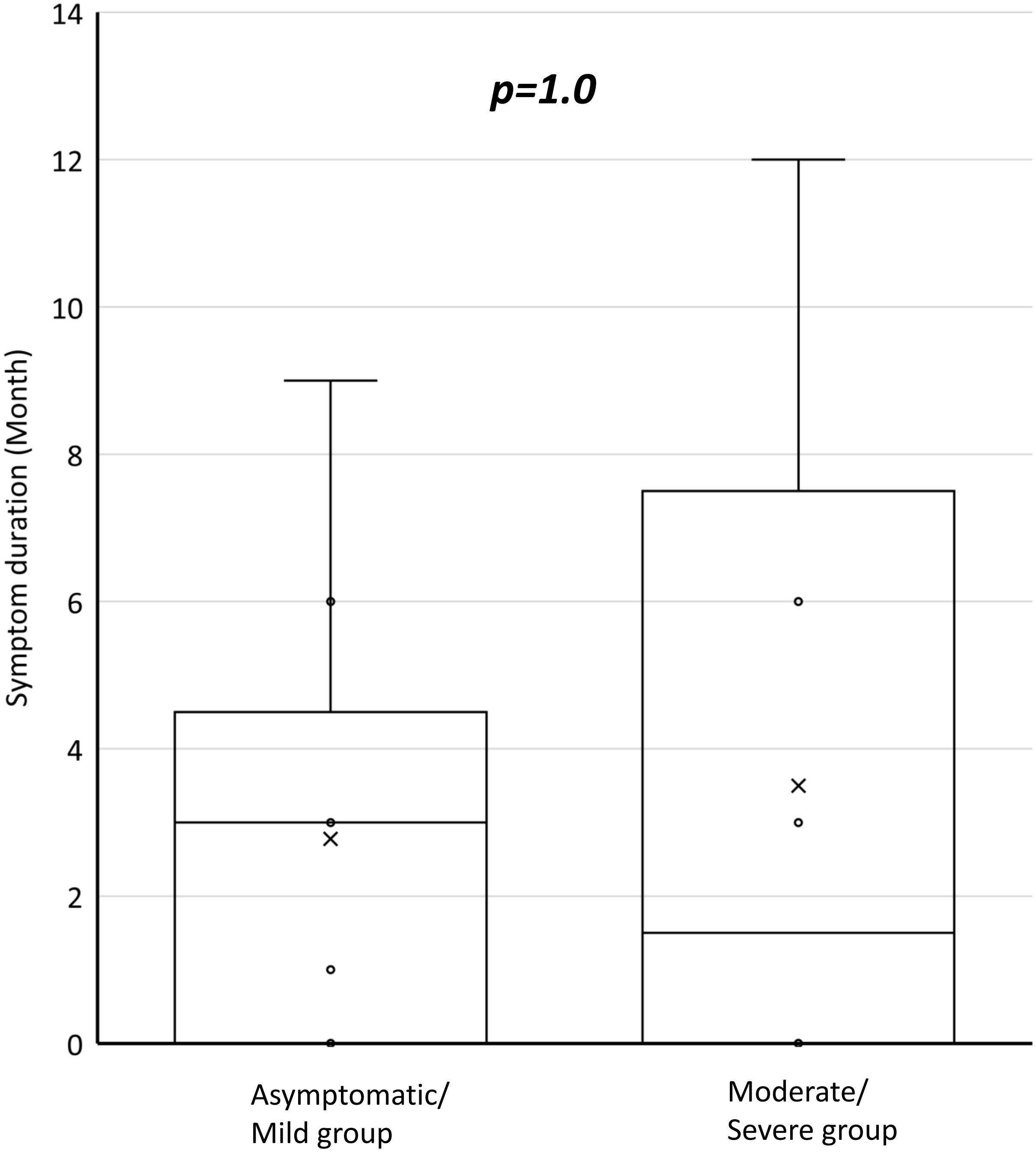

Figure Legend:
Supplemental figure 1

Symptom duration was compared between Asymptomatic/ Mild group and Moderate/ Severe group. The duration of the most prolonged symptoms from each participant was compared. There were no statistically significant differences by the Mann-Whitney U-test.

Supplemental figure 2

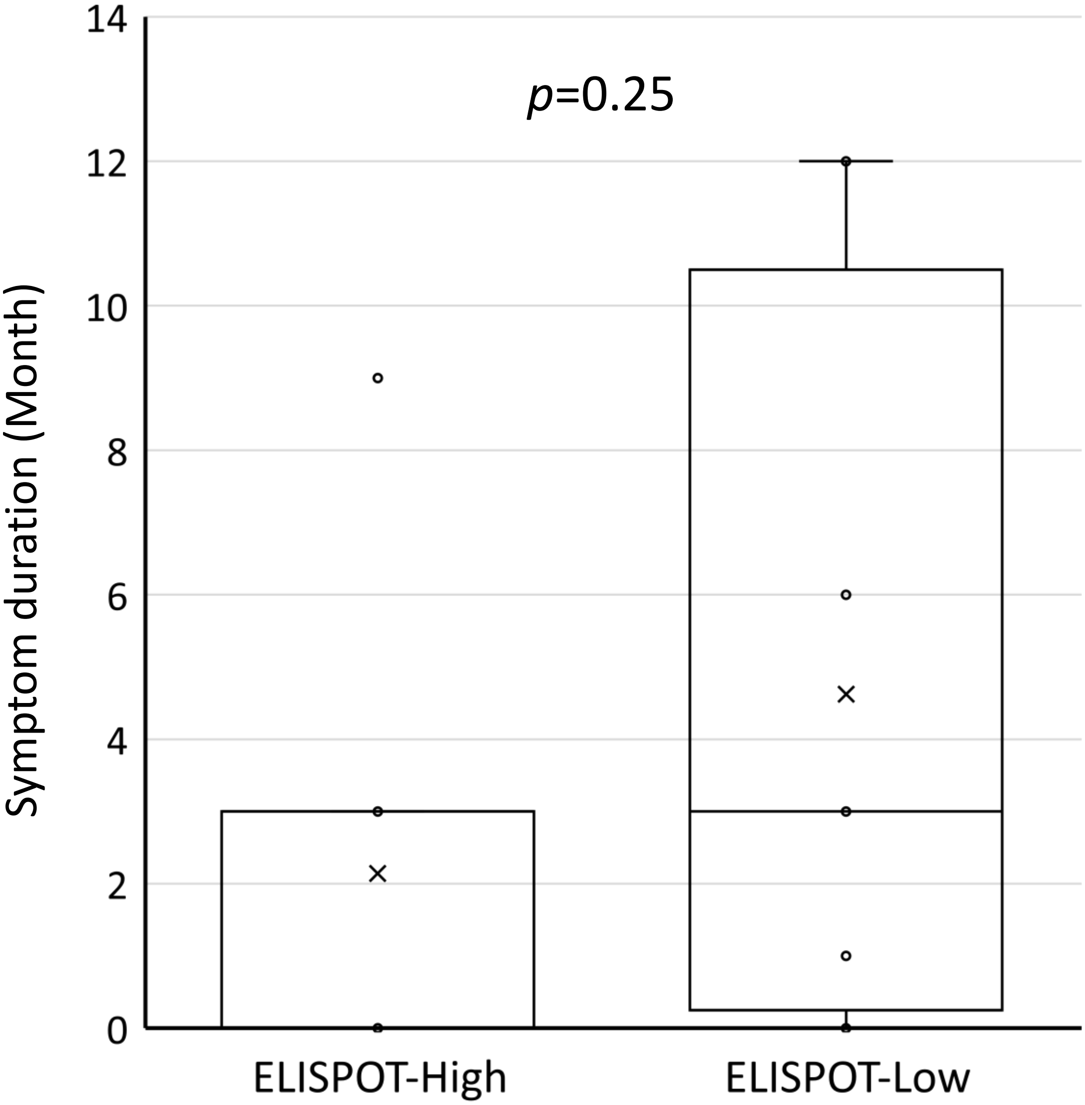

Figure Legend:
Supplemental figure 2

Symptom duration was compared between ELISPOT-high group and low group. The duration of the most prolonged symptoms from each participant was compared. Although there were no statistically significant differences by the Mann-Whitney U-test, ELISPOT-low group showed a tendency for symptoms to persist for a longer period.

Supplemental figure 3

	ELISPOT-high (n=7)	ELISPOT-low (n=8)	
	Median (Q1-Q3)		*p* value
Sampling (Days after diagnosis)	40 (35 - 46)	43 (38 - 117)	*p*=0.61

Figure Legend:
Supplemental figure 3

The comparison of the number of days from diagnosis to the blood sampling between ELISPOT-high and -low group. The result showed no significant difference between those two groups with a p-value of 0.61 by the Mann-Whitney U test.
